# Suspension culture in a T-flask with acoustic flow induced by ultrasonic irradiation

**DOI:** 10.1016/j.ultsonch.2021.105488

**Published:** 2021-02-10

**Authors:** Genichiro Fujii, Yuta Kurashina, Yusuke Terao, Tetsushi Azuma, Akira Morikawa, Kazuhide Kodeki, Osamu Takahara, Kenjiro Takemura

**Affiliations:** a School of Science for Open and Environmental Systems, Graduate School of Science and Technology, Keio University, Japan; b Department of Mechanical Engineering, Faculty of Science and Technology, Keio University, Japan; c Department of Materials Science and Engineering, School of Materials and Chemical Technology, Tokyo Institute of Technology, Japan; d Mitsubishi Electric Corporation, Japan

**Keywords:** Suspension culture, Acoustic streaming, Ultrasonic, Cell proliferation, Chinese hamster ovary, Cell activity

## Abstract

Suspension culture is an essential large-scale cell culture technique for biopharmaceutical development and regenerative medicine. To transition from monolayer culture on the culture surface of a flask to suspension culture in a bioreactor, a pre-specified cell number must first be reached. During this period of preparation for suspension culture, static suspension culture in a flask is generally performed because the medium volume is not large enough to use a paddle to circulate the medium. However, drawbacks to this static method include cell sedimentation, leading to high cell density near the bottom and resulting in oxygen and nutrient deficiencies. Here, we propose a suspension culture method with acoustic streaming induced by ultrasonic waves in a T-flask to create a more homogeneous distribution of oxygen, nutrients, and waste products during the preparation period preceding large-scale suspension culture in a bioreactor. To demonstrate the performance of the ultrasonic method, Chinese hamster ovary cells were cultured for 72 h. Results showed that, on average, the cell proliferation was improved by 40% compared with the static method. Thus, the culture time required to achieve a 1000-fold increase could be reduced by 32 h (a 14% reduction) compared with the static method. Furthermore, the ultrasonic irradiation did not compromise the metabolic activity of the cells cultured using the ultrasonic method. These results demonstrate the effectiveness of the ultrasonic method for accelerating the transition to large-scale suspension culture.

## Highlights

• Exposure to 158 kHz ultrasound enables suspension culture without damaging cells.
• Cell proliferation was 40% higher than that with the conventional method after 72 h.
• This technique accelerates the transition to large-scale suspension culture.

## 1 Introduction

The development of biopharmaceuticals to treat serious illnesses is growing, and this type of pharmaceutical accounts for about 30% of prescription and over-the-counter drug sales. More than half of the 100 pharmaceuticals with the highest sales in 2018 were biopharmaceuticals [1]. The production of biopharmaceuticals relies on cultured cells, and about 70% of all biopharmaceuticals are produced by mammalian cells such as the Chinese hamster ovary (CHO) cell line [2]. The culture of mammalian cells is also important in regenerative medicine [3], [4]. A single treatment for severe myocardial infarction requires 1 × 10^8^ to 1 × 10^9^ cardiomyocytes differentiated from induced pluripotent stem cells [5]. Thus, large-scale cell culture is crucial to efficiently produce biopharmaceuticals and regenerative medicine-based treatments.

**Fig. 1:**
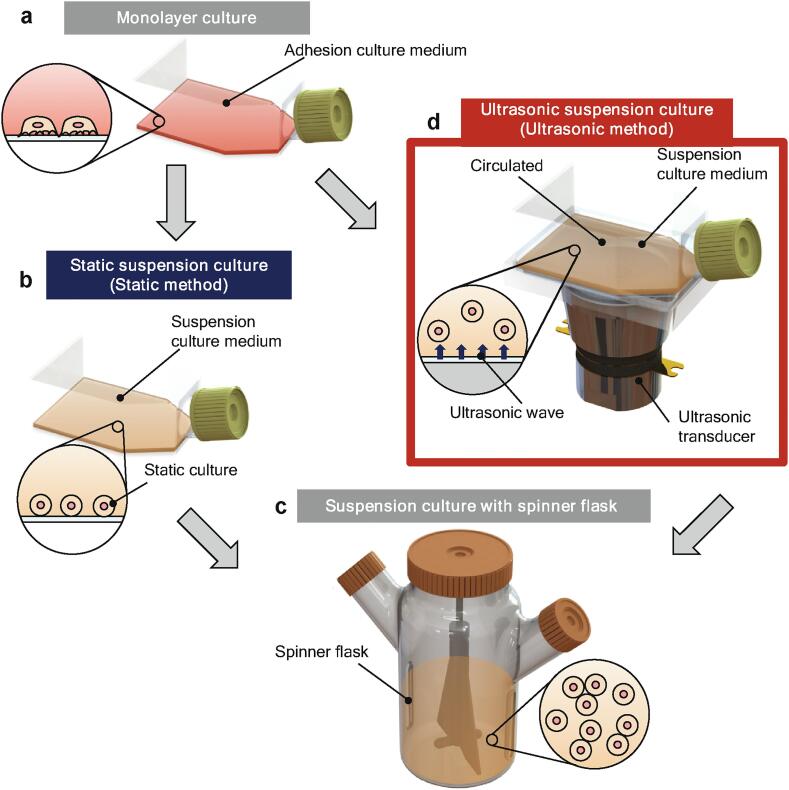
Sequential cell culture process for large-scale culture of adherent cells. (a) Adherent cells are cultured on the culture surface of a cell culture flask with adherent cell culture medium. (b) To acclimate the cells to the suspension culture medium and increase the number of cells, cells are statically cultured in a cell culture flask with suspension culture medium (herein referred to as the static method). (c) Once the cell number has increased, the suspended cells are cultured in spinner flasks. (d) The ultrasonic method, which can serve as an alternative to the static method for acclimating the cells to the suspension culture medium and increasing the number of cells—the cells are cultured in suspension culture medium and exposed to ultrasonic waves generated by an ultrasonic irradiation device.

In the large-scale culture of adherent cells, cells are first developed in a small-scale monolayer culture on the culture surface of a flask [6] (Fig. 1a). However, monolayer culture provides poor volumetric efficiency because the cells must attach to a culture surface. Furthermore, in large-scale culture with high cell numbers, the medium should be stirred continuously to distribute the oxygen, nutrients, and waste products. The most common large-scale bioreactors include a simple glass container with a stirring element for stirring the medium [7], a rotating-wall vessel bioreactor, and a cylindrical vessel that rotates slowly about its horizontal axis [8], [9]; the spinner flask is one of the most well-known bioreactors for the mass culture of adherent cells. While multi-layer culture techniques have been ultrasonic to improve the volumetric efficiency [10], [11], suspension culture is the most common method for large-scale culture because it offers volumetric efficiency and provides a homogeneous distribution of oxygen, nutrients, and waste products. However, a large number of cells are needed to stir the cell suspension; even in a small spinner flask (4500–125, Corning Inc, NY, USA), more than 2 × 10^7^ cells/125 mL is required. Thus, the cells must be further proliferated following monolayer culture (10^4^–10^5^ cells) before they can be transferred to a spinner flask (which requires 10^7^ or more cells). Therefore, once the cell number has increased in monolayer culture, the cells are first transferred to static suspension culture with a flask, in which cells are seeded in a culture medium suitable for suspension culture (Fig. 1b) [6], [12]; then, once the number of cells in the static suspension culture has further increased, the cells are transferred to a bioreactor (Fig. 1c). However, this conventional static suspension culture step is inefficient and can be rate-limiting.

In this study, we fabricated an ultrasonic irradiation system with a Langevin ultrasonic transducer and demonstrated an effective ultrasonic suspension culture method (Fig. 1d) to replace static suspension culture in the transition from monolayer culture to spinner flask culture. Herein, we evaluate the potential of the ultrasonic method for large-scale cell culture by investigating its efficiency in terms of the improvement in cell proliferation compared with the standard culture method and confirming that the technique does not damage cells.

## 2 Materials and methods

### 2.1 Preparation of cells

The CHO cell line (CHO-K1, RCB0403, Riken Bio Resource Center, Ibaraki, Japan) was used as a representative cell line, as CHO cells are commonly used for protein production [2], including protein-based biopharmaceuticals. For the monolayer culture, CHO cells were cultured on the culture surface of a T-flask (Nunc Cell Culture T25 EasyFkask, Thermo Fisher Scientific, MA, USA) in Ham’s F-12 medium (α-MEM Ham’s F-12, Wako, Tokyo, Japan) supplemented with 10% fetal bovine serum (FBS, S1820, Biowest SAS, Nuaillé, France) in a 5% CO_2_ humidified incubator at 37 °C.

### 2.2 Cell culture method

The cells (2.0 × 10^6^) prepared as described above were cultured in PF-CHO medium (10 mL, HyQ PF-CHO MPS, GE Healthcare, IL, USA) supplied with sodium hydroxide and L-glutamine in an incubator with 5% CO_2_ at 37 °C for 3 days using either the conventional static suspension culture method or the ultrasonic suspension culture proposed in this study. In the static method [6], the CHO cells were suspended in PF-CHO medium on the culture surface of the T-flask (Nunc Non-treated T25 EasyFlask, Thermo Fisher Scientific, MA, USA) in a 5% CO_2_ humidified incubator at 37 °C. In the ultrasonic method, ultrasonic waves were applied to the culture surface of the T-flask using an ultrasonic irradiation device to stir the medium and cells.

### 2.3 Conditions for optimization experiments

The effect of the ultrasonic method on the cell culture efficiency was evaluated with nine different conditions: all combinations of culture medium volume (3, 5, and 10 mL) and seeded cell number (2 × 10^4^, 2 × 10^5^, and 2 × 10^6^ cells). The cells to be seeded were counted using a cell counter (TC20 Automated Cell Counter, Bio-Red Laboratories, CA, USA). After the culture period, living cells were distinguished from dead cells by staining the dead cells with Trypan blue (Trypan Blue Solution 0.4%, Thermo Fisher Scientific) to evaluate the effect of the ultrasonic irradiation on cell viability. The doubling time, *T*_d_, was calculated by Hayflick’s formula as follows:

(1) *r* = (3.32 log *X*_2_/*X*_1_)/*t*

(2) *T*_d_ = 1/*r*

where *r*, *X*_1_, *X*_2_ and *t* are the multiplication rate, the initial cell number, the cell number at the end of culture and culture time, respectively.

### 2.4 Cell growth assay

Two rounds of cell culture were conducted successively to further verify differences in cell proliferation under different culture conditions. The cells were seeded in a T-flask and cultured using the ultrasonic method or the static method for 72 h. Then, the cells were re-seeded and re-cultured using the same method for another 72 h. The numbers of live and dead cells were counted using the cell counter, and cell growth curves were drawn using the results from both cultures and fitting exponential approximation curves to the data for each culture condition.

### 2.5 LDH assay

A lactate dehydrogenase (LDH) assay was used to estimate the extent of natural and induced cell damage in order to evaluate the cytotoxicity of the ultrasonic method. Four cell suspensions were prepared: two sample suspensions, a maximum LDH suspension, and a blank suspension. The sample suspensions were cell-free supernatants from centrifuged cell cultures prepared with each method. The maximum LDH suspension was the supernatant collected from a cell suspension in which all cells were killed with Triton X-100 (Sigma-Aldrich, MO, USA). The blank suspension contained only cell-free culture medium.

Next, 50 μL of each prepared assay suspension and working solution (CK12 Cytotoxicity LDH Assay Kit-WST, Dojindo, Tokyo, Japan) were mixed in a 96-well plated and incubated for 30 min at room temperature. Then, 25 μL of stop solution was added to each well to stop the reaction. The absorbance at 490 nm was measured in each well using a plate reader (Multiskan FC, Thermo Fisher Scientific). The cytotoxicity, *P*, of the ultrasonic method was then calculated using measured absorbance values from the four suspensions as follows:

(3) $$P=\frac{\left(\lambda_{A}-\lambda_{D}\right)-\left(\lambda_{B}-\lambda_{D}\right)}{\left(\lambda_{C}-\lambda_{D}\right)-\left(\lambda_{B}-\lambda_{D}\right)}\times 100,$$

where *λ*_A_, *λ*_B_, *λ*_C_, and *λ*_D_ were the absorbances of the sample suspensions from the ultrasonic method and the static method, the maximum LDH suspension, and the blank suspension, respectively.

### 2.6 Cell proliferation assay

The cell proliferation was evaluated after the culture by each method. Cells were cultured by the ultrasonic method or the static method, then independently re-seeded at a concentration of 2 × 10^6^ cells in 10 mL of culture medium and again cultured under the same conditions. Then, the cells were re-seeded in T-flask and incubated for 72 h. The cells were then counted using the cell counter, and the cell proliferation rate of the cells cultured with the ultrasonic method was compared with that of the cells cultured with the static method.

### 2.7 Glucose consumption assay

The glucose consumption of the re-seeded cells was measured to evaluate the cell metabolism after culturing by each method. The supernatant medium was sampled after 72 h in culture, and cell-free medium was prepared as a blank. A total of 10 µL of the sample and 200 µL of assay reagent from an assay kit (Glucose (HK) Assay Kit, Sigma-Aldrich, MO, USA) were mixed in a 96-well plate and incubated for 15 min at room temperature. After the reaction, the absorbance at 340 nm was measured using a plate reader. Glucose consumption, *C*, was calculated as follows:

(4) $$C=\frac{V_{total}∙\left(\lambda_{blank}-\lambda_{sample}\right)}{\varepsilon ∙d∙V_{sample}∙N_{P}}$$

where *λ*_blank_ and *λ*_sample_ are absorbances of the blank and the sample, respectively, *ε* represents the millimolar extinction coefficient at 340 nm for NADH (the reduced form of nicotinamide adenine dinucleotide), *d* represents the light path, *N*_p_ represents the number of cells, and *V*_total_ and *V*_sample_ are the volumes of total assay suspension and the sample, respectively.

### 2.8 Lactate production assay

A lactate production assay was conducted to evaluate the cell metabolism. The same supernatant medium samples used for the glucose consumption assay were used for the lactate production assay. First, the assay reagent was prepared by combining lactate production assay buffer, lactate enzyme mix, and probe provided in the lactate production assay kit (Lactate Colormetric/Fluorometric Assay Kit, BioVision Inc., CA, USA) at a 23:1:1 ratio. The samples were prepared by mixing 0.5 µL of medium sample with 24.5 µL of the lactate production assay buffer in a 96-well plate and incubating for 30 min at room temperature and protected from light. The absorbance at 570 nm was measured from each well using a plate reader, and the lactate production was estimated using the prepared standard curve.

### 2.9 Statistics and reproducibility

Differences between groups of samples were evaluated using analysis of variance (ANOVA) with Ryan’s multiple comparison test. Values of *p* < 0.05 (denoted as *) or *p* < 0.01 (denoted as **) were considered statistically significant.

## 3 Results

### 3.1 Fabrication and vibration characteristics of the ultrasonic irradiation device

**Fig. 2:**
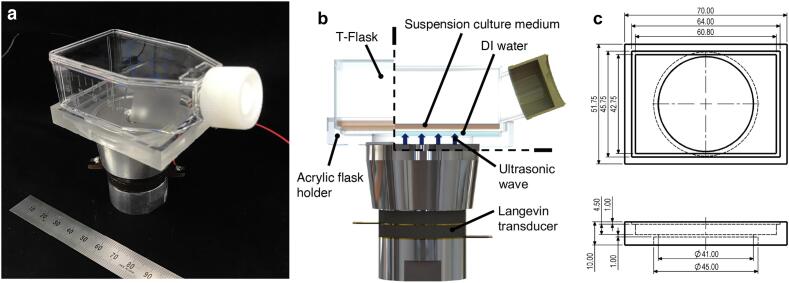
Ultrasonic irradiation device for suspension cell culture. (a) Photograph of the ultrasonic irradiation device and (b) side view of the experimental setup, where the dashed lines in (b) delineate a cross-section view. (c) Dimension of acrylic flask holder.

The ultrasonic irradiation device (Fig. 2a) comprised a 25 cm^2^ T-flask, an acrylic flask holder, and a Langevin ultrasonic transducer (HEC-45402, Honda Electronics, Tokyo, Japan). Note that to suppress the occurrence of cell-damaging cavitation [13], [14], a transducer with a resonance frequency higher than the dozens of kHz commonly used was employed. Resonant vibration of a transducer was induced by excitation with a function generator (WF1946B, NF Corporation, Kanagawa, Japan) and an amplifier (HAS4051, NF Corporation); the resulting ultrasonic waves were applied to the culture medium from the culture surface of the T-flask, as shown in Fig. 2b. The flask holder (Fig. 2c) was filled with sterilized distilled (DI) water that allowed the ultrasonic waves to propagate the culture medium within the flask. Note that the ultrasonic waves were effectively transmitted to the flask since the intensity transmission coefficient of the ultrasonic wave [15] was 0.95 by using DI water as an intermediary.

**Fig. 3:**
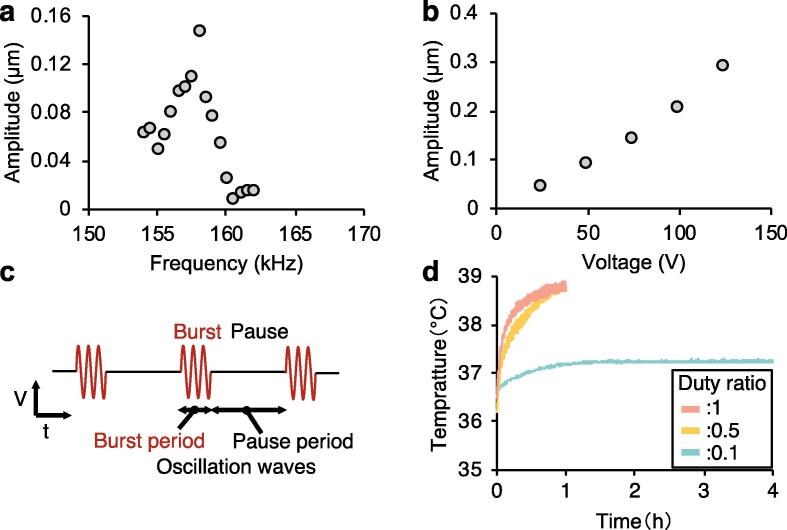
Characteristics of the ultrasonic irradiation device with a Langevin piezoelectric transducer. (a) Relationship between the frequency and vibration amplitude around the resonance frequency when the input voltage was 75 V_p-p_. (b) Relationship between the input voltage and maximum amplitude with a driving frequency of 158 kHz. (c) The intermittent burst waves of ultrasonic irradiation (Input voltage (V) vs duration time (t)). (d) Thermal history of the intermittent burst waves of the ultrasonic irradiation device. The driving frequency was 158 kHz, the vibration amplitude was 0.15 µm. The medium was exposed to repeated cycles of ultrasonic irradiation for 30 s, followed by no irradiation for 90 s. The burst period and the pause period for each duty ratio of 1, 0.5, and 0.1 were 1000 and 0, 500 and 500, and 100 and 900 oscillation waves, respectively.

The vibration characteristics of the transducer were evaluated to determine the input frequency and vibration amplitude for suspension culture. The amplitude of the excited vibration was measured at the center of the transducer using a laser Doppler vibrometer (LV-1800, ONO Sokki, Kanagawa, Japan) with 75 V_p-p_ as the input voltage. Fig. 3a shows the relationship between the frequency and vibration amplitude. Based on the increase and decrease in the amplitude, the resonance frequency was determined to be 158 kHz. Fig. 3b shows the relationship between the input voltage and vibration amplitude at the 158 kHz resonance frequency. The vibration amplitude increased linearly with increasing input voltage. Hence, the input frequency was set to the resonance frequency (158 kHz) for the subsequent experiments.

### 3.2 Thermal history

The heat generated from ultrasonic irradiation is a serious problem that may negatively impact cell viability [13]. To ensure that the cells can be cultured with an appropriate temperature (37 °C) with the ultrasonic method, we measured the temperature during ultrasonic irradiation. The output vibration conditions were as described above (frequency: 158 kHz; vibration amplitude: 0.15 µm; input voltage: 75 V_p-p_). The ultrasonic irradiation was applied as intermittent bursts to mitigate the heat generation (Fig. 3c) with the duty ratio of 1, 0.5, and 0.1. The burst period and the pause period for each duty ratio were 1000 and 0, 500 and 500, and 100 and 900 oscillation waves, respectively. The cells were repeatedly exposed to ultrasonic irradiation in cycles of 30 s followed by no irradiation for 90 s. The temperature of 10 mL medium in a T-flask was measured using a thermometer (tr-71wf, T&D Corporation, Nagano, Tokyo) under these ultrasonic irradiation conditions. Fig. 3d shows the thermal history of the medium during ultrasonic wave irradiation in a 5% CO_2_ humidified incubator. With the duty ratios of 1 and 0.5, the temperature raised to nearly 39 °C after 1 h, respectively. On the other hand, once the temperature reached around 37 °C with the duty ratio of 0.1, the temperature was maintained for more than 3 h. These results indicate that these irradiation conditions and this pattern of intermittent bursts (duty ratio: 0.1) are suitable to conduct cell culture experiments.

### 3.3 Medium volume and number of seeded cells for optimal culture conditions

**Fig. 4:**
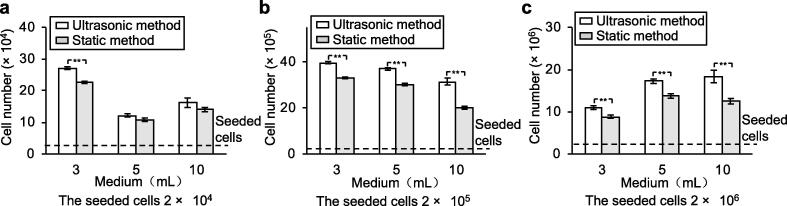
Comparison of the number of living cells with various medium volumes and numbers of seeded cells. The cells were cultured using the ultrasonic method and the static method with medium volumes of 3, 5, and 10 mL for 72 h. The initial numbers of seeded cells were (a) 2 × 10^4^, (b) 2 × 10^5^, and (c) 2 × 10^6^ cells (bars represent the mean ± SD, *n* = 4, **: *p* < 0.01).

Next, we experimentally evaluated different initial cell concentrations for the proposed ultrasonic suspension culture. CHO-K1 cells were cultured with the three different initial cell concentrations and three different medium volumes with a frequency of 158 kHz, vibration amplitude of 0.15 µm, and input voltage of 75 V_p-p_. Fig. 4 shows the relationship between the medium volume and numbers of living cells after 72 h in culture with different initial cell numbers. Under all initial cell concentrations, the number of cells was higher after 72 h in culture with the ultrasonic method than with the static method; this difference was statistically significant under seven of the nine conditions. The doubling time (*T*_d_) is shown in Table 1. As these results show that the cell culture efficiency was improved under all initial cell concentrations, subsequent experiments were conducted with 2.0 × 10^6^ cells in 10 mL of culture medium.

**Table 1:** Doubling time of cells cultured by ultrasonic and static suspension culture.

	

### 3.4 Cell growth evaluation

**Fig. 5:**
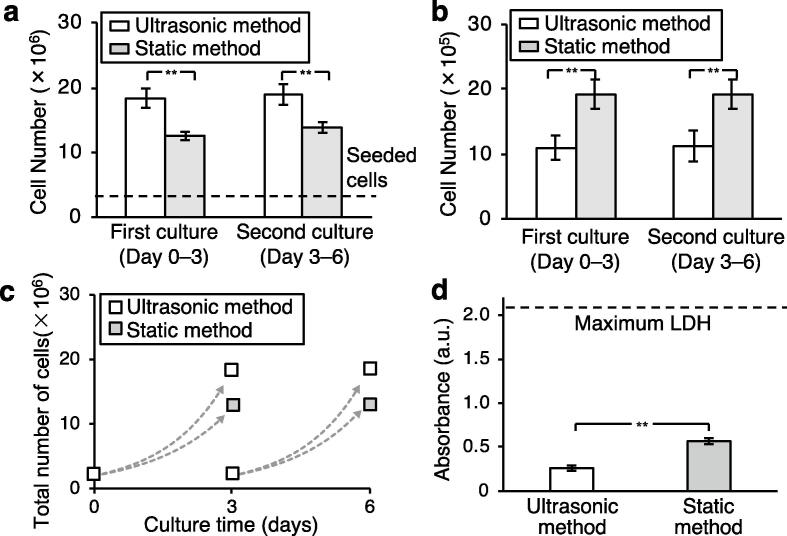
Comparison of the numbers of living and dead cells and LDH absorbance in the medium. (a) Comparison of the number of living cells. Cells were cultured for 72 h (days 0–3) using the ultrasonic method and the static method, then subsequently re-cultured for 72 additional hours (days 3–6) by the same method. The number of seeded cells was 2 × 10^6^. (b) Comparison of the number of dead cells after culturing for 72 h. (c) Growth curves of living cells during the first and second culture periods using the ultrasonic method and the static method. The growth curves of both methods were approximated by exponential curves. (d) Comparison of LDH absorbances in the two sample solutions derived from cultures with the ultrasonic method and the static method (bars show the mean ± SD, *n* = 4, **: *p* < 0.01).

To evaluate the cell culture efficiency of continuous culture with the ultrasonic method, we counted the number of living or dead cells after culture by the ultrasonic method and static method. Furthermore, to confirm the repeatability of the experimental results, the cells were propagated through a second culture period under the same conditions. In other words, the first 3 day culture (days 0–3) was followed by a second 3 day culture (days 3–6). Fig. 5a and b show the numbers of living and dead cells after the first and second stages of culture, respectively, with each method. After the first stage of culture (days 0–3), the number of living cells was 1.47 times higher, and the number of dead cells was 0.57 times lower, with the ultrasonic method (*T*_d_: 22.5 h) compared with the static method (*T*_d_: 27.2 h). Similarly, after the second stage of culture (days 3–6), the number of living cells was 1.37 times higher, and the number of dead cells was 0.58 times lower, with the ultrasonic method (*T*_d_: 22.2 h) compared with the static method (*T*_d_: 25.9 h). Fig. 5c shows the growth curves during the first and second culture stages. The growth curves of successive stages of culture were similar with the ultrasonic method.

**Fig. 6:**
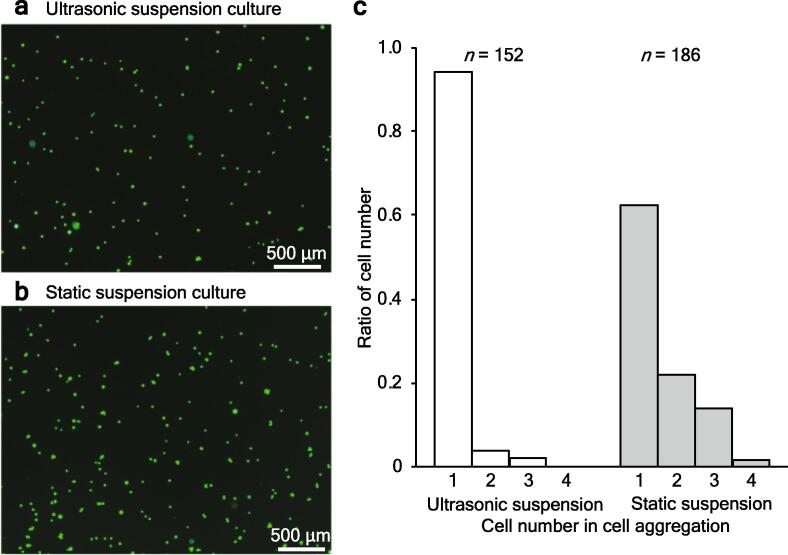
CHO cells after three days of culture were observed. (a,b) Fluorescence images of cultured cells by (a) ultrasonic suspension culture and (b) static suspension culture. Cells were stained with calcein AM. (c) Ratio of cell aggregation in ultrasonic and static suspension culture.

Cell cytotoxicity was estimated using the LDH assay. Fig. 5d shows the absorbance of the supernatant medium from the cultures with both methods relative to that of the maximum LDH solution (i.e., the LDH value when all cells were killed). The cytotoxicity, *P* (calculated using Eq. (3)), of the ultrasonic method was 22% lower than that of the static method. This result indicates that the cells are less damaged by the ultrasonic method than the static method. In addition, to evaluate the condition of the cultured cells, the cells were observed after three days of culture. The cells cultured with the ultrasonic method (Fig. 6a) and the static method (Fig. 6b) were stained with calcein AM (C0875, Sigma-Aldrich, MO, USA) and observed by a fluorescence microscope. The number of cells per cell aggregation is shown in Fig. 6c. The results show that ultrasonic suspension culture prevents cell aggregation.

### 3.5 Cell viability and metabolism

**Fig. 7:**
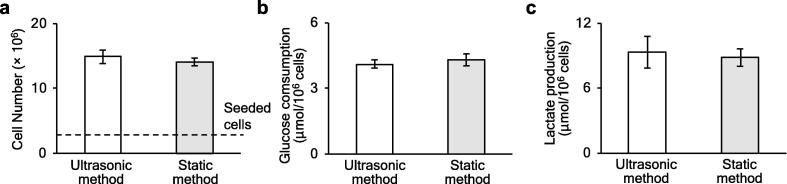
Effect of ultrasonic waves on cell viability and metabolism. (a) Number of living cells after re-culturing with the statistic culture method for 72 h following initial culture by the ultrasonic method or static method. The number of initially seeded cells was 2 × 10^6^. (b) Glucose consumption in the medium where cells were re-cultured for 72 h after culturing by the ultrasonic or static method for 72 h. (c) Lactate production in the medium where cells were re-cultured for 72 h after culturing by the ultrasonic method or static method for 72 h (bars show the mean ± SD, *n* = 4).

To evaluate the persistent effect of ultrasonic irradiation on cell proliferation and metabolism, we compared the cell proliferation, glucose consumption, and lactate dehydrogenase activity after 72 h in culture using the ultrasonic method versus the static method followed by re-culturing with the static method in a T-flask. Fig. 7a shows the cell proliferation during the 72 h static suspension culture following the initial culture with each method; no statistically significant difference was observed. To evaluate the effect of ultrasonic irradiation on cell metabolism, the glucose consumption and lactate production activity were measured during static suspension culture for 72 h following initial culture with the ultrasonic method or the static method. In the metabolism of glycolysis, 2 mol of lactate is produced from l mol of glucose. Fig. 7b and c shows that the amounts of produced lactate (9.3 and 8.9 µmol per 10^6^ cells in the ultrasonic and static methods, respectively) were about twice the corresponding amounts of consumed glucose (4.1 and 4.3 µmol per 10^6^ cells in the ultrasonic and static methods, respectively). This finding suggests that metabolism proceeded normally under both methods. There were no statistically significant differences between the glucose consumption or lactate production activity in the ultrasonic method compared with the static method. As no abnormalities were observed in the proliferation or metabolism of the cells after culture by the ultrasonic method, it was concluded that ultrasonic radiation under these conditions did not affect cell proliferation or metabolism.

## 4 Discussion

In this study, we demonstrated an ultrasonic suspension culture technique as an efficient suspension culture method (Fig. 1d) to facilitate the transition from monolayer culture (Fig. 1a) to large-scale suspension culture in a bioreactor, such as a spinner flask (Fig. 1c). Experimental results showed that cell proliferation and cell metabolism were not inhibited by the ultrasonic irradiation involved in this culture method.

Initiating a large-scale culture in a bioreactor requires a large number of cells, which can be time-consuming and rate-limiting. Therefore, it is desirable to minimize the time required to reach the target cell number in the intermediate stage between the monolayer culture and the suspension culture in a spinner flask. The time needed to increase the cell number from 2 × 10^4^ to the cell number required for suspension culture in a spinner flask (2 × 10^7^) can be calculated based on the growth curves obtained in this study (Fig. 4, Fig. 5). Because cells proliferated by repeated cell divisions, the relationship between cell number and cell division is given by

(5) $$N_{72h}={N_{initial}\times 2}^{P_{72h}},$$

where *N*_initial_ represents the initial number of cells, *P*_72h_ represents the number of cell divisions in 72 h, and *N*_72h_ represents the number of cells proliferated after 72 h. The relationship between *N*_initial_ and the number of cell divisions required to achieve a ten-fold increase in cell number is as follows:

(6) $${N_{initial}\times 2}^{P_{10times}}=10\times N_{initial}.$$

Therefore, the culture time, *t*, required to proliferate cells to a 10-fold increase in cell number is given by

(7) $$t=72h/P_{72h}\times P_{10times}=72h\times \log_{2}10/\log_{2}\frac{N_{72h}}{N_{initial}}.$$

By inputting the experimental data into Eq. (7), the culture time required for the number of cells to increase ten-fold under the tested culture conditions was calculated. The *t* value with the ultrasonic method was calculated to be 63.6, 60.2, and 74.8 h when *N*_inital_ was 2 × 10^4^, 2 × 10^5^, and 2 × 10^6^ cells, respectively, and the medium volume was 3, 10, was 10 mL, respectively, while those with the static method were 68.4, 71.9, and 90.5 h, respectively. Hence, the culture time required to increase the cell number from 2 × 10^4^ to 2 × 10^7^ is estimated to be 198.6 h with the ultrasonic method and 230.7 h with the static method. Thus, the ultrasonic method is expected to decrease the total culture time by 32 h, which is equivalent to 14% of the total time.

Experimental results showed that the number of cells was greater after 72 h of culture with the ultrasonic method than that with the static method. The difference was observed under various medium volumes and cell seeding numbers, and the differences were statistically significant in seven of the nine conditions tested (Fig. 4). This finding suggests that the ultrasonic method is effective over a wide range of conditions. The two conditions that did not show statistically significant differences were 5 mL medium with 2 × 10^4^ seeded cells and 10 mL medium with 2 × 10^4^ seeded cells, which may be attributed to the relatively low cell density compared with the other conditions. Cell proliferation is known to be significantly lower when the cell density is low [16]. Therefore, although the proliferation rate with the ultrasonic method was higher than that with the static method over 72 h in culture, the difference may have been too small to be statistically significant. Among the conditions in which the ultrasonic method resulted in significantly higher increases in cell number than the static method, the cell proliferation rate varied slightly, suggesting that there is an optimal amount of medium for each number of seeded cells.

In the static method, cells generally accumulate on the culture surface of the T-flask [17], leading to a high local cell density and insufficient oxygen and nutrient supplies to the cells. As a result, the cell proliferation rate decreases. The ultrasonic waves used in the ultrasonic method were expected to induce circulation and better distribute the oxygen and nutrients in the culture. In the development of the ultrasonic irradiation device, it was necessary to consider whether ultrasound could be safely applied to the cells. Critical factors that may cause cell damage include the cavitation associated with ultrasonic waves and heat arising from the output energy of the ultrasonic waves balanced with heat radiation [13], [18]. The cavitation was mitigated by providing intermittent burst waves of ultrasound irradiation [19]. Furthermore, the temperature was measured during intermittent burst waves of ultrasound irradiation to ensure that it did not exceed around 37 °C (Fig. 3C), which is considered suitable for cell culture. In addition, experimental results showed that the ultrasound irradiation did not affect the growth curve, proliferation, or metabolism, further indicating that the cells were not damaged (Fig. 4, Fig. 5, Fig. 6).

Other cell actuation technologies based on ultrasonic irradiation have been developed and used to detach [20], [21], [22], collect [23], and manipulate [24] cells without any damage arising from various phenomena such as ultrasonic waves, ultrasonic pressure, acoustic streaming, or standing wave trapping. In the ultrasonic irradiation device developed in this study, the ultrasonic waves may induce acoustic radiation forces [25], a standing wave between the transducer and the medium surfaces [26], and acoustic streaming from the attenuation of sound pressure [27]. However, the acoustic radiation force is expected to be extremely small because the acoustic impedance of a cell (about 1.60 × 10^6^ kg m^-2^s^−1^ [28]) is about the same as that of the cell culture medium (about 1.55 × 10^6^ kg m^-2^s^−1^ [28]). Additionally, a standing wave cannot be formed in this system because the distance between the transducer and the medium surface (approximately 1.2, 2.0, and 4.0 mm for medium volumes of 3, 5, and 10 mL, respectively) is shorter than the wavelength in the medium (approximately 9.5 mm). Hence, the cells should be circulated by acoustic streaming in this system.

This acoustic streaming-induced stirring function can be performed without any contact, resulting in reduced risk of contamination. The experimentally derived growth curves (Fig. 5a and c) and LDH activity levels (Fig. 5d) suggest that cell activity was improved in the ultrasonic method compared with the static method. Our findings confirm that the ultrasonic method improves the cell proliferation rate compared with the static method without affecting cell activity. Another possible stirring method is the suspension culture using an orbital shaker; however, the ultrasonic suspension method has several advantages not found in the orbital shaker method. Since a flask itself does not need to be shaken like the orbital shaker method and the swirling force does not change depending on the location of flask, the culture system may be compact in size. In addition, the orbital shaker is prone to swirling sloshing, which causes cells to aggregate at the center of flask [29], [30] depending on the amount of medium and the swirling speed. On the other hand, swirling sloshing is hardly caused in the ultrasonic suspension culture because the medium is stirred from the bottom to the top. Hence, the use of ultrasonic waves is a promising technique for improving and accelerating the preparation of cells in monolayer culture to transition to large-scale culture. This technique may also be suitable for culturing blood cells [31] and mononuclear cells [32], which naturally proliferate in suspension.

## 5 Conclusion

We developed an ultrasonic suspension culture method in which cells can be stirred by the acoustic streaming induced by the ultrasonic irradiation. We ran a series of the ultrasonic and static suspension cultures using CHO cells as a model example, with varying culture conditions, in order to demonstrate the effectiveness of the ultrasonic suspension culture in a small amount of medium. The main finding is that the cell proliferation was improved compared with the conventional static suspension culture. Accordingly, the culture time of the ultrasonic suspension culture required to increase the number of cells 1000-fold was reduced by 14% compared to the static suspension culture. The increased proliferation is attributed to most of the cells being able to maintain a single-cell state during culture. Suspension culture is an important technology for the mass culture required in biopharmaceutical development and regenerative medicine, and this study could be a breakthrough to accelerate the efficiency of the initial stage of suspension culture.

## CRediT authorship contribution statement

**Genichiro Fujii:** Methodology, Validation, Investigation, Writing - original draft. **Yuta Kurashina:** Conceptualization, Methodology, Validation, Formal analysis, Investigation, Writing - original draft. **Yusuke Terao:** Methodology, Investigation. **Tetsushi Azuma:** Resources, Funding acquisition. **Akira Morikawa:** Resources, Funding acquisition. **Kazuhide Kodeki:** Resources, Funding acquisition. **Osamu Takahara:** Resources, Funding acquisition. **Kenjiro Takemura:** Conceptualization, Writing - review & editing, Supervision.

## Declaration of Competing Interest

The authors declare the following financial interests/personal relationships which may be considered as potential competing interests: Authors have applied for a patent (PCT number: PCT/JP2018/025577) related to this manuscript.
